# Transkulturelle deutschsprachige Übersetzung des Fragebogens Reflux Symptom Score-12

**DOI:** 10.1007/s00106-022-01233-2

**Published:** 2022-10-14

**Authors:** Johanna Bruhn, M. Brockmann-Bauser, Tyler Swing, Jörg E. Bohlender, Daniel Runggaldier

**Affiliations:** 1 Klinik für Otorhinolaryngologie, Head and Neck Surgery, Abt. Phoniatrie und Klinische Logopädie, Universitätsspital Zürich, Frauenklinikstrasse 24, 8091 Zürich, Schweiz; 2 Universität Zürich, Rämistrasse 71, 8006 Zürich, Schweiz; 3 Gemeinschaftspraxis Wiesendangen, Wiesendangen, Schweiz

**Keywords:** Laryngopharyngealer Reflux, Symptomfragebogen, Reflux Symptom Index, German Version des Reflux Symptom Score-12, Refluxdiagnostik, Laryngopharyngeal reflux, Symptom questionnaire, Reflux symptom index, German version of the reflux symptom score-12, Reflux diagnostics

## Abstract

Bei der Diagnostik des laryngopharyngealen Refluxes (LPR) spielen neben einer anamnestischen und klinischen bzw. apparativen Beurteilung auch Fragebögen zur detaillierten Erfassung der Symptomatik eine wesentliche Rolle. Neben dem Reflux Symptom Index (RSI), dem bekanntesten LPR-Symptomfragebogen, wurde kürzlich auch ein neuer Fragebogen, „Reflux Symptom Score“ (RSS), sowie eine kürzere Version, der Reflux Symptom Score 12 (RSS-12), veröffentlicht. Letzterer ist allerdings nur in englischer, französischer und portugiesischer Sprache verfügbar, ermöglicht jedoch eine wesentlich genauere Differenzierung der Symptomatik unter Einbeziehung von Symptomstärke, Häufigkeit des Auftretens von Beschwerden sowie der refluxbezogenen Beeinträchtigung der Lebensqualität. Das Ziel dieser Arbeit ist daher, eine entsprechende transkulturelle deutschsprachige Übersetzung des RSS-12 (aktuell nun als G‑RSS-12 bezeichnet) mit Verständlichkeitstestung vorzustellen, um den klinischen und wissenschaftlichen Einsatz dieses Fragebogens auch im deutschsprachigen Raum zu ermöglichen.

## Abstract (en)

In addition to clinical and instrumental methods as well as medical history, questionnaires are well-established tools in the diagnosis of laryngopharyngeal reflux (LPR). Recently, the reflux symptom score (RSS) as well as the short version of it, the RSS-12, which are self-administered patient-reported outcome questionnaires, have been published as an alternative to the well-established reflux symptom index (RSI). The RSS-12 assesses severity and frequency of LPR symptoms as well as the impact on quality of life and has been shown to have high reliability and validity. However, so far, only French, English, and Portuguese versions of the RSS-12 have been published. Hence, the aim of this study was to prepare a transcultural German translation of the RSS-12 (G-RSS-12) to provide a new clinical and scientific tool for the diagnosis of LPR in German-speaking regions.

Zur Diagnostik des laryngopharyngealen Refluxes (LPR), der durch den Rückfluss von gastroduodenalem Sekret oder durch Aufstoßen von Gasen aus dem Magen bis in den Larynx- und Pharynxbereich charakterisiert ist, wird aktuell eine Vielzahl an unterschiedlichen Untersuchungsmethoden angewandt [6]. Neben einer detaillierten Anamnese, den endoskopischen Verfahren, aber auch den neueren pH-metrischen Verfahren oder Pepsin-Speichelmessungen besitzen auch Fragebögen zur detaillierten Erfassung der Symptomatik einen wesentlichen klinischen und wissenschaftlichen Stellenwert. Dabei ist der Reflux Symptom Index (RSI) der älteste und auch bekannteste Symptomfragebogen mit insgesamt 9 Items [2]. Trotz intensiver Validierung und des Einsatzes in vielen Studien haben sich hier in den letzten Jahren zahlreiche Schwachstellen gezeigt. So sind die im RSI aufgelisteten Symptome unspezifisch und werden oftmals auch im Rahmen von diversen anderen Krankheitsbildern, wie beispielsweise oberen Atemwegsinfekten oder chronischen Rhinosinusitiden ohne pH-metrisch bestätigten Reflux, beklagt [4, 11]. Ebenfalls finden Parameter wie Symptomhäufigkeit, aber auch die Beeinträchtigung der Lebensqualität, im RSI keine Berücksichtigung [11].

Ein kürzlich durch das „international project of young otolaryngologists of the International Federation of Otorhinolaryngological Societies“ etablierter neuer „Reflux Symptom Score“ (RSS) versucht eine wesentlich genauere Differenzierung der Symptomatik unter Einbeziehung von Symptomstärke, Häufigkeit des Auftretens von Beschwerden sowie der Beeinträchtigung der Lebensqualität vorzunehmen. Auch wurde dieser Fragebogen bereits in einer englischen, französischen und koreanischen Version publiziert und validiert [7, 8, 10]. Aufgrund der Länge des RSS mit insgesamt 22 Items wurde zur Vereinfachung für den klinischen Alltag auch eine gekürzte Fassung des RSS publiziert und validiert (Tab. 1). Dieser als Reflux Symptom Score-12 (RSS-12) bezeichnete Fragebogen existiert zwar in einer englischen, französischen und portugiesischen Version [3, 8], eine deutsche Übersetzung dieses Fragebogens ist bisher jedoch noch nicht veröffentlicht worden. Das Ziel dieser Arbeit war es daher, eine entsprechende deutschsprachige Übersetzung des RSS-12 (nun als G‑RSS-12 bezeichnet) zu erstellen, um den klinischen und wissenschaftlichen Einsatz dieses Fragebogens auch im deutschsprachigen Raum zu ermöglichen.

**Table 1:** 

Reflux Symptom Score-12			
Within the last month, I suffered from one/several of the following symptoms Frequency: 0 = I don’t have this complaint over the past month (sic!), 1;2;3;4 = I had 1–2; 2–3; 3–4; 4–5 weekly over the past month; 5 = complaint occurs daily Severity: 0 = problem is not severe, 5 = problem very troublesome when it occurs			
**Ear, nose and throat disorders**	**Disorder frequency**	**Disorder severity**	**Quality of life impact**
Hoarseness or a voice problem	0‑1-2-3-4‑5	0‑1-2-3-4‑5	0‑1-2-3-4‑5
Throat pain or pain during swallowing	0‑1-2-3-4‑5	0‑1-2-3-4‑5	0‑1-2-3-4‑5
Difficulty swallowing (pills, liquids or solid foods)	0‑1-2-3-4‑5	0‑1-2-3-4‑5	0‑1-2-3-4‑5
Throat clearing (not cough) (sic!)	0‑1-2-3-4‑5	0‑1-2-3-4‑5	0‑1-2-3-4‑5
Sensation of something being stuck in the throat	0‑1-2-3-4‑5	0‑1-2-3-4‑5	0‑1-2-3-4‑5
Excess mucous in the throat and/or post nasal drip sensation	0‑1-2-3-4‑5	0‑1-2-3-4‑5	0‑1-2-3-4‑5
Bad breath	0‑1-2-3-4‑5	0‑1-2-3-4‑5	0‑1-2-3-4‑5
Heartburn, stomach acid coming up, regurgitation, burping, or nausea	0‑1-2-3-4‑5	0‑1-2-3-4‑5	0‑1-2-3-4‑5
Abdominal pain or diarrhea	0‑1-2-3-4‑5	0‑1-2-3-4‑5	0‑1-2-3-4‑5
Indigestion, abdominal distension and/or flatus	0‑1-2-3-4‑5	0‑1-2-3-4‑5	0‑1-2-3-4‑5
Coughing (not just throat clearing)	0‑1-2-3-4‑5	0‑1-2-3-4‑5	0‑1-2-3-4‑5
Breathing difficulties, breathlessness, or wheezing	0‑1-2-3-4‑5	0‑1-2-3-4‑5	0‑1-2-3-4‑5
	RSS total score:		Quality of life score:

## Methoden

Der gekürzte und ins Englische übersetzte RSS-12-Fragebogen [7, 9] stellte die Ausgangsversion unserer Übersetzung dar (Tab. 1). Mit einer 6 Punkte umfassenden Likert-Skala werden die Häufigkeit und Schwere der Symptomatik evaluiert. Für jedes einzelne Item werden die Wertungen für Häufigkeit und Schwere der Symptomatik multipliziert und anschließend miteinander addiert. Daraus ergibt sich der RSS-12-Score. Ergebnisse über 11 werden als pathologisch gewertet [7]. Zusätzlich wird die Beeinträchtigung der Lebensqualität durch das jeweilige Item erhoben, ein Einfließen dieser in die Berechnung des RSS-12-Score findet jedoch nicht statt. Die vorliegende Übersetzung wurde methodisch nach dem Ablauf für transkulturelle Übersetzungen nach Beaton [1] in fünf Schritten durchgeführt.

Im ersten Schritt wurden die deutschen Übersetzungen T‑1 durch eine Fachperson sowie T‑2 durch eine fachfremde Person erstellt und die damit einhergehenden Schwierigkeiten protokolliert. Im anschließenden zweiten Schritt wurde aus den Übersetzungen T‑1 und T‑2 unter Berücksichtigung der protokollierten Schwierigkeiten die Syntheseversion T‑12 verfasst. Der dritte Schritt umfasste die Rückübersetzung durch eine Fachperson und eine fachfremde Person, beide englische Muttersprachler, welche den ursprünglichen Fragebogen nicht kannten. Dabei entstanden die Rückübersetzungen BT‑1 und BT‑2 mit den jeweiligen Übersetzungsprotokollen. Diese wurden im vierten Schritt in einem Expert*innenkomitee gemeinsam evaluiert. Dabei wurden Diskrepanzen diskutiert und die Übersetzung hinsichtlich ihrer semantischen, idiomatischen, konzeptuellen und Erfahrungsäquivalenz überprüft. Der Abgleich folgte dabei sowohl mit der englischen Version als auch der französischen Originalversion. Auf Basis dieser Diskussion und Überprüfungen entstand der präfinale Fragebogen (nun als G‑RSS-12 bezeichnet). Das Expert*innenkomitee bestand aus PD Dr. med. J. Bohlender, Leiter der Abteilung für Phoniatrie und Klinische Logopädie der Klinik für Ohren‑, Nasen‑, Hals- und Gesichtschirurgie des Universitätsspitals Zürich, PD Dr. phil. M. Brockmann-Bauser, Fachleiterin Klinische Logopädie und Leiterin der Forschungsgruppe Phoniatrie und Klinische Logopädie, Dr. med. D. Runggaldier sowie med. pract. Johanna Bruhn.

Im fünften und letzten Schritt wurde der präfinale Fragebogen G‑RSS-12 an zehn Personen unterschiedlichen Alters und Geschlechts im persönlichen Umfeld der Autoren auf Verständlichkeit geprüft. Diese wurde nach Ausfüllen des Fragebogens in Interviews mittels eines semistandardisierten Fragebogens in Anlehnung an die „EORTC Quality of Life Group Translation Procedure“ durchgeführt [5]. Dabei beantworten die Proband*innen Fragen zu jedem Item hinsichtlich Schwierigkeiten, die bei der Beantwortung der Frage gegebenenfalls aufgetreten waren. Die Rückmeldungen wurden ausgewertet. Basierend auf den Rückmeldungen wurden entsprechende Anpassungen und Optimierungen im Layout durchgeführt.

## Ergebnisse

Bei der Verständlichkeitstestung umfasste die Proband*innengruppe jeweils 5 Frauen und 5 Männer ohne relevante Vorerkrankungen im Alter zwischen 23 und 86 Jahren. Die häufigsten Angaben (*n* = 2) von Schwierigkeiten beim Verständnis traten bei den Items 8, 10 und 12 auf. Diese wurden jeweils als „verwirrend“ beschrieben. So wurden die Begriffe „Verdauungsprobleme“ (Item 10) und „Bauchschmerzen“ (Item 9) als sehr ähnlich und damit als redundant betrachtet. Auch bei Item 10 (Atembeschwerden, Luftnot oder Atemgeräusch) fiel es Proband*innen zum Teil schwer, die einzelnen Begriffe zu differenzieren.

**Figure 1:**
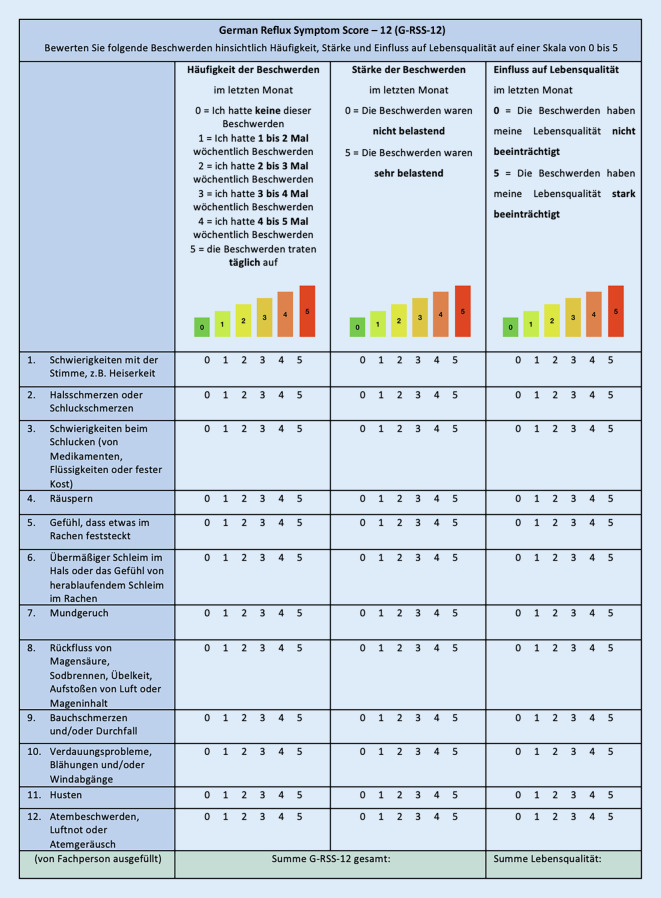

Zudem gaben 3 Proband*innen an, dass sie die letzte Zeile, die der Auswertung dient, als verwirrend empfanden. Wir ergänzten daher eine Beschreibung, dass der Score von einer Fachperson berechnet und ausgefüllt werden soll. Da 3 Testpersonen zusätzlich berichteten, ihnen sei es schwergefallen, bei den drei Bewertungskategorien und der 6‑Punkte-Likert-Skala den Überblick zu behalten, wurde das Layout des G‑RSS-12 um eine Farbcodierung ergänzt. Der transkulturell übersetzte und optimierte Fragebogen G‑RSS-12 ist in Abb. 1 dargestellt.

## Diskussion

Bei der Diagnostik des LPR nehmen Fragebögen neben der klinischen Untersuchung sowie der klinisch-apparativen Diagnostik einen wichtigen Stellenwert ein, insbesondere auch im Rahmen einer Screeningfunktion. Trotz eingehender Validierung und des Einsatzes in Forschungsprojekten und im klinischen Alltag haben sich aufgrund von fehlenden Parametern wie Symptomhäufigkeit oder der Beeinträchtigung der Lebensqualität zahlreiche Einschränkungen und Schwachstellen in Bezug auf den bislang gängigen Reflux Symptom Index (RSI) herauskristallisiert [6, 8].

Der Reflux Symptom Score-12 (RSS-12), der eine wesentlich genauere Differenzierung der LPR-Symptomatik unter Einbeziehung von Symptomstärke, Häufigkeit des Auftretens von Beschwerden sowie der Beeinträchtigung der Lebensqualität berücksichtigt, stellt somit einen neuen und möglicherweise vielversprechenden Fragebogen dar. Bislang ist dieser nur in englischer, französischer und portugiesischer Sprache veröffentlicht worden [3, 8], sodass sich die vorliegende Arbeit der transkulturellen Übersetzung des RSS-12 in die deutsche Sprache widmet.

Im Expert*innenkomitee erfolgte sowohl ein Abgleich mit der englischsprachigen Fassung als auch der französischsprachigen Originalversion. Im Expert*innenkomitee wurde der Fragebogen hinsichtlich fachlicher und sprachlicher Äquivalenz sowie Verständlichkeit geprüft. Schwierigkeiten, die die Übersetzer*innen protokollierten, betrafen vor allem den Begriff der „post nasal drip sensation“, für den es auf Deutsch keine allgemein verständliche Übersetzung gibt. Insbesondere für die Verständlichkeit für medizinische Laien wurde daher die deskriptive Beschreibung „Gefühl von herablaufendem Schleim im Hals“ gewählt.

In der anschließenden Verständlichkeitsprüfung zeigte sich ein allgemein gutes Verständnis der deutschen Übersetzung des RSS-12. Die Anmerkungen von 2 Proband*innen bezogen sich hier vor allem auf das Vorkommen ähnlicher Begriffe. Änderungen der präfinalen Version nach der Befragung betrafen dann lediglich das Layout und das Einfügen von Erläuterungen**.**

Der RSS-12 basiert auf dem RSS, welcher jedoch nicht unumstritten ist. In der Kritik steht dieser unter anderem in Bezug auf seine Validität und Auswahl der Items [12]. Die Validierung der französischsprachigen Version des RSS-12 zeigte jedoch eine angemessene interne Konsistenz, im Vergleich zum RSI eine hohe externe Validität und eine hohe Retest-Reliabilität [8]. Zudem konnte eine hohe Inhaltsvalidität des RSS-12 im Vergleich zu anderen patientenzentrierten Feedbackmethoden gezeigt werden [7], sodass parallel zur Übersetzung der englischen Version im Expert*innengremium auch ein Abgleich mit der französischsprachigen Version durchgeführt wurde.

Insgesamt kann dem G‑RSS-12 daher Potenzial zugeschrieben werden, sich in Bezug auf den LPR als hilfreiches Tool in Forschung und Klinik zu etablieren. Mit der deutschsprachigen Übersetzung des RSS-12 besteht nun eine Möglichkeit, die initiale wie auch die Verlaufssymptomatik des LPR im deutschsprachigen Raum strukturiert zu erfassen, wobei gleichzeitig auch Informationen über die Beeinträchtigung der Lebensqualität gewonnen werden können. Dies könnte auch für die jeweiligen weiteren Therapieentscheidungen von Nutzen sein. Einschränkend gilt jedoch, dass eine Validierung des G‑RSS-12 noch ausstehend ist. Dies sollte daher bei der Anwendung in Klinik und Forschung entsprechend berücksichtigt werden.

## Fazit für die Praxis

- Fragebögen nehmen neben der klinischen und apparativen Untersuchung einen wichtigen Stellenwert bei der Diagnostik des LPR ein.
- Neben dem bisher eingesetzten Reflux Symptom Index (RSI) könnte sich der nun auf Deutsch übersetzte Reflux Symptom Score 12 (G-RSS-12) als wichtiges Werkzeug zur Erfassung der Häufigkeit und Schwere der LPR-Symptomatik und der damit einhergehenden Einschränkung der Lebensqualität etablieren.
- Eine weiterführende Validierung des deutschsprachigen G‑RSS-12 steht noch aus.
